# Adsorption of SARS‐CoV‐2 Spike Protein S1 at Oxide Surfaces Studied by High‐Speed Atomic Force Microscopy

**DOI:** 10.1002/anbr.202000024

**Published:** 2020-12-18

**Authors:** Yang Xin, Guido Grundmeier, Adrian Keller

**Affiliations:** ^1^ Technical and Macromolecular Chemistry Paderborn University Warburger Str. 100 Paderborn 33098 Germany

**Keywords:** Al_2_O_3_, biointerfaces, high-speed atomic force microscopy, SARS-CoV-2, TiO_2_

## Abstract

The ongoing coronavirus disease 2019 (COVID‐19) pandemic caused by the severe acute respiratory syndrome coronavirus 2 (SARS‐CoV‐2) represents a serious threat to the health of millions of people. Respiratory viruses such as SARS‐CoV‐2 can be transmitted via airborne and fomite routes. The latter requires virion adsorption at abiotic surfaces and most likely involves the SARS‐CoV‐2 spike protein subunit 1 (S1), which is the outermost point of its envelope. Understanding S1 spike protein interaction with fomite surfaces thus represents an important milestone on the road to fighting the spread of COVID‐19. Herein, high‐speed atomic force microscopy (HS‐AFM) is used to monitor the adsorption of the SARS‐CoV‐2 spike protein S1 at Al_2_O_3_(0001) and TiO_2_(100) surfaces in situ. While the single‐crystalline oxide substrates are chosen to model the native surface oxides of Al‐ and Ti‐based fomites, adsorption is studied in electrolytes that mimic the pH and major ionic components of mucosal secretions and saliva, respectively. Quantitative analysis of the obtained HS‐AFM images indicates that S1 spike protein adsorption at these surfaces is mostly governed by electrostatic interactions with possible contributions from van der Waals interactions. It thus proceeds more rapidly at the TiO_2_(100) than at the Al_2_O_3_(0001) surface.

## Graphical abstract

The adsorption of the severe acute respiratory syndrome coronavirus 2 (SARS‐CoV‐2) spike protein subunit 1 (S1) at Al_2_O_3_ and TiO_2_ surfaces is investigated under electrolyte conditions mimicking different bodily fluids by high‐speed atomic force microscopy. Quantitative image analysis reveals that S1 protein adsorption at these two fomite surfaces is driven by electrostatic interactions with possible contributions of van der Waals interactions.

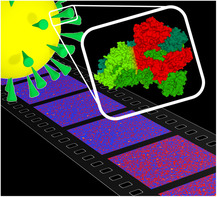

## 1 Introduction

Since its first appearance in December 2019 in Wuhan, China, coronavirus disease 2019 (COVID‐19) has spread around the globe with cases observed in virtually every country on earth. On December 14, 2020, more than 70 million cases were reported worldwide with global deaths exceeding 1.6 million.^[^ ^1^ ^]^ Caused by the severe acute respiratory syndrome (SARS) coronavirus 2 (SARS‐CoV‐2), the current COVID‐19 pandemic represents the third epidemic outbreak of a previously unknown zoonotic coronavirus in less than 20 years. The 2003 SARS outbreak resulted in more than 8000 infections and 774 confirmed deaths, while Middle East respiratory syndrome (MERS) killed 858 of the 2494 people who contracted the illness between 2012 and 2019.^[^ ^2^ ^]^

Respiratory viruses such as SARS‐CoV‐2 can be transmitted among humans via four main routes: short‐range airborne transmission via droplets, long‐range airborne transmission via aerosols, direct contact transmission via human–human contact, and indirect contact transmission via contaminated intermediate objects (fomites).^[^ ^3^ ^]^ Although numerous respiratory disease outbreaks have been studied to identify the relative importance of the individual transmission routes, fundamental knowledge on transmission routes that could be used to improve intervention strategies is still missing.^[^ ^3^ ^]^ This is particularly true for fomite transmission, which has not been considered a possible transmission route at all up until the 1980s.^[^ ^4^ ^]^ Nowadays, however, growing evidence suggests that contaminated surfaces play a key role in the spread of viral infections.^[^ ^4^ ^]^ While it is generally accepted that coronaviruses such as SARS‐CoV‐1, SARS‐CoV‐2, and MERS‐CoV are predominantly transmitted via the airborne routes,^[^ ^5^ ^]^ several studies identified fomite transmission as a major factor in several coronavirus outbreaks, in particular in healthcare settings.^[^ ^6^ ^]^ However, little is known about the physicochemical mechanisms of the interactions of these viruses with abiotic surfaces and how nonspecific virus‐surface interactions affect virus viability and infectiousness. Such knowledge will be particularly important not only with regard to the development of antiviral coatings but also for adapting sterilization and disinfection protocols during epidemic outbreaks when shortages of protective gear and disinfectants are experienced by healthcare personnel as it was the case in Europe during the first months of the COVID‐19 pandemic.

**Figure 1:**
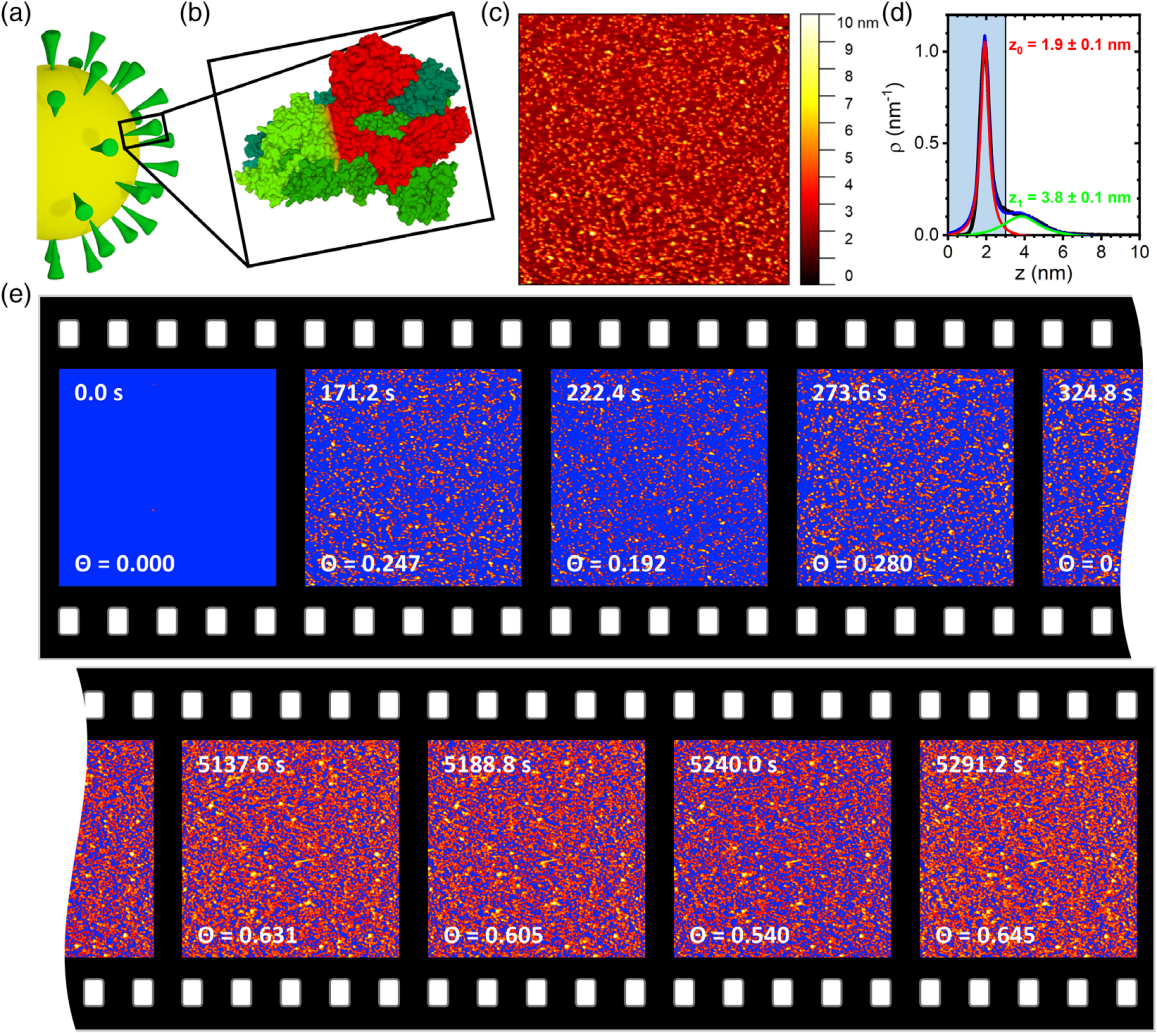
a) Schematic representation of SARS‐CoV‐2. b) Molecular structure of the SARS‐CoV‐2 spike protein trimer (PDB ID 6VXX, from RCSB PDB, rcsb.org).^[^ ^9^ ^]^ The S1 domain of one monomer is highlighted in red. c) HS‐AFM image (1 × 1 μm^2^) of SARS‐CoV‐2 spike protein S1 (5 μg mL^−1^ in 10 mM Tris at pH 7.5) on an Al_2_O_3_(0001) surface after incubation for about 171.2 s. d) Corresponding height distribution function. The substrate (red) and protein peaks (green) have been fitted with Lorentzian functions. The vertical line represents the height threshold applied in the statistical analysis, with the shaded area indicating the substrate region. e) Consecutive HS‐AFM images of the adsorption of SARS‐CoV‐2 spike protein S1 (5 μg mL^−1^ in 10 mM Tris at pH 7.5) at an Al_2_O_3_(0001) surface recorded in the beginning and the end of the experiment. The HS‐AFM images have been thresholded based on the individual height distribution functions to separate the substrate from the protein peaks as exemplified in panel (d). For the pristine Al_2_O_3_(0001) surface without protein at 0.0 s, a height threshold similar to the other surfaces (3 nm) was applied. All pixels in the substrate region below the threshold are indicated in blue. The respective time points and calculated protein surface coverage Θ are given in the images.

A few recent studies investigated the viability of SARS‐CoV‐2 viruses on different abiotic surfaces in laboratory settings and found that adsorbed virions may remain active for hours to days depending on the surface material.^[^ ^7^, ^8^ ^]^ So far, however, there are no molecular‐level investigations of the adsorption of SARS‐CoV‐2 virions at fomite surfaces. In this work, we thus study the adsorption of the SARS‐CoV‐2 spike protein subunit 1 (S1) in a selection of relevant electrolytes at Al_2_O_3_ and TiO_2_ surfaces. The spike protein S1 is not only involved in the specific interaction of SARS‐CoV‐2 virions with biotic surfaces,^[^ ^9^ ^]^ but due to its position in the viral envelope also represents the first point of contact in the nonspecific adsorption at abiotic surfaces (see **Figure** **1**a,b). Because of the high demand and low expression rate of the recombinant S1 spike protein, however, only comparatively low amounts are commercially available, which renders adsorption studies with established techniques such as quartz crystal microbalance^[^ ^10^, ^11^ ^]^ or ellipsometry^[^ ^11^, ^12^ ^]^ rather challenging. Therefore, we turned to high‐speed atomic force microscopy (HS‐AFM) instead, which enables the visualization of the adsorption, diffusion, and interaction dynamics of various biomolecules at biotic and abiotic surfaces in situ and in real time.^[^ ^13^, ^14^, ^15^, ^16^ ^]^ As this approach requires ultrasmooth surfaces, we used single‐crystalline Al_2_O_3_(0001) and TiO_2_(100) substrates as model surfaces for the native oxide films on Al‐ and Ti‐based fomites.

## 2 Results and Discussion

Figure 1c shows an in situ HS‐AFM image of an Al_2_O_3_(0001) surface in contact with 5 μg mL^−1^ SARS‐CoV‐2 spike protein S1 dissolved in 10 mM Tris at pH 7.5. Individual protein particles can clearly be resolved. The corresponding height distribution function is shown in Figure 1d. Here, a dominant narrow peak is observed at 1.9 nm, corresponding to the substrate surface. The presence of adsorbed proteins results in a small secondary peak at 3.8 nm. The low intensity of this peak relative to the substrate peak indicates the comparatively low surface coverage. Based on previous cryoelectron microscopy investigations, the S1 spike protein has an anisotropic 3D shape (see Figure 1b) with the major axes having dimensions of about 10 and 15 nm, respectively.^[^ ^9^ ^]^ The comparatively low height of the adsorbed protein of only about 2 nm may thus be somewhat surprising. While proteins often undergo conformational changes during adsorption,^[^ ^17^ ^]^ such large height differences are less frequent. Nevertheless, adsorption‐induced height changes in a similar magnitude have previously been observed, for instance, for serum albumin and thyroglobulin adsorbed at titanium oxide and silicon oxide surfaces.^[^ ^17^ ^]^ However, it should also be noted that the dimensions of the S1 subunit mentioned earlier correspond to the fully assembled spike protein trimer (see Figure 1b). In the absence of the other protein components, the isolated and monomeric S1 subunit may adopt a different conformation with different dimensions.

**Figure 2:**
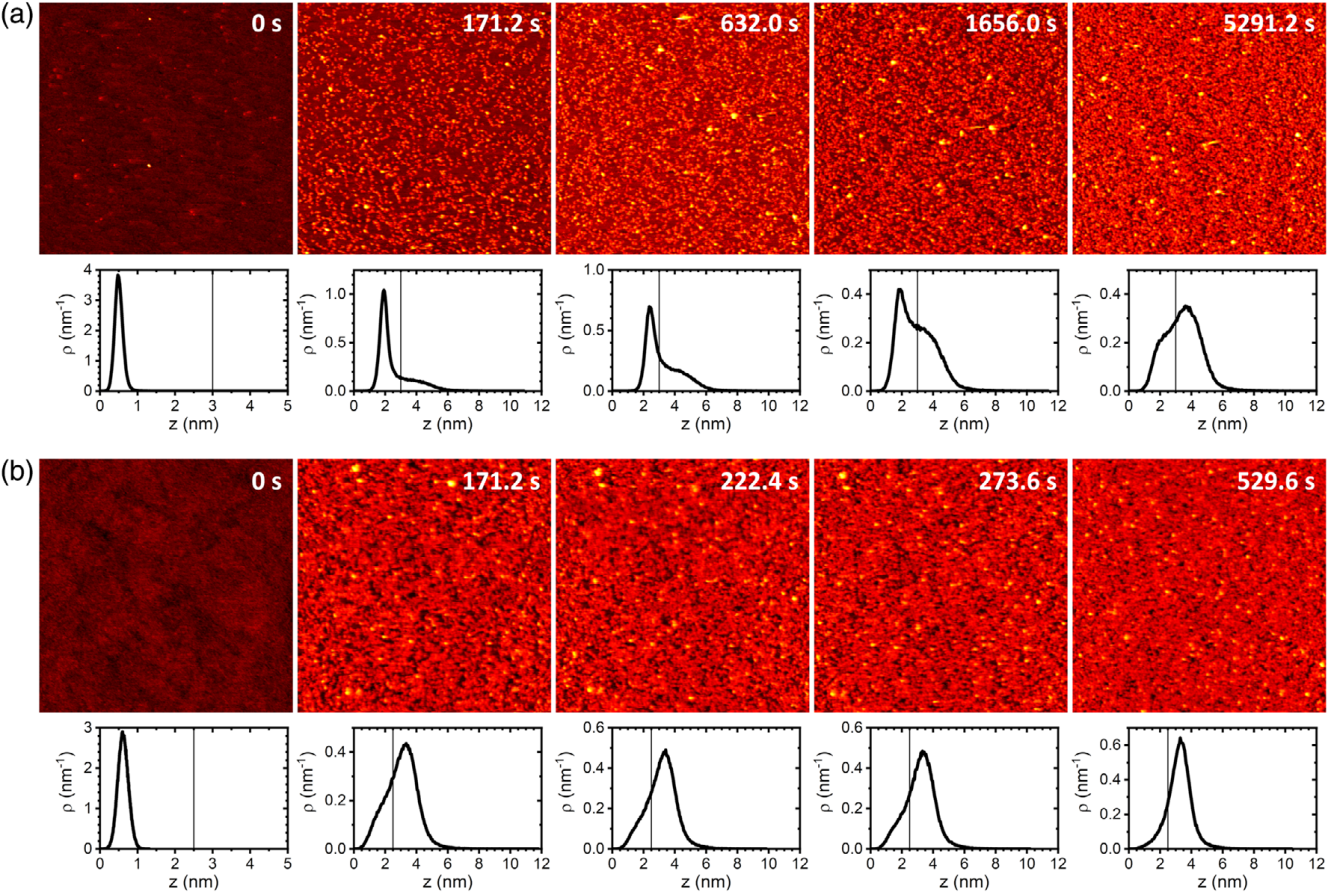
HS‐AFM images (1 × 1 μm^2^) of SARS‐CoV‐2 spike protein S1 in 10 mM Tris (pH 7.5) adsorbed to a) an Al_2_O_3_(0001) and b) a TiO_2_(100) surface recorded at different time points as indicated. Height scales are 5 nm for the clean substrate surfaces at 0 s and 12 nm for the protein covered surfaces at later time points. Below the HS‐AFM images, the corresponding height distribution functions are depicted. The vertical lines in the plots represent the height thresholds applied in the statistical analyses.

To assess adsorption dynamics, S1 spike protein adsorption under these conditions has been monitored by HS‐AFM over close to 90 min. HS‐AFM enables the monitoring of biomolecular dynamics with a temporal resolution down to about 100 ms per frame.^[^ ^18^ ^]^ However, at such high frame rates, the rapidly scanned tip may interfere with biomolecular motion and binding and thus result in serious scanning‐induced artifacts.^[^ ^15^, ^16^ ^]^ Furthermore, as several previous studies have shown, many biomolecular systems have much slower kinetics that can be captured already with frame rates of the order of several seconds per frame.^[^ ^14^, ^19^ ^]^ Therefore, in this work, we chose a scan rate of 51.2 s per frame. Under these scan conditions, no tip effects could be detected (see Figure S4, Supporting Information), while S1 spike protein adsorption could be followed with sufficient detail. **Figure** **2**a shows selected HS‐AFM images recorded at different time points. As can be seen, more and more proteins are adsorbing with increasing incubation time. This can also be observed in the corresponding height distribution functions. Here, the secondary peak is becoming more intense with time. After 5291.2 s of incubation, this protein peak has become the dominant contribution. Nevertheless, the surface peak can still be observed in the form of a shoulder at small height values, which is an evidence that the surface is not fully covered with the protein films even after such a long incubation time.

To quantitatively assess SARS‐CoV‐2 spike protein adsorption at the Al_2_O_3_(0001) surface, the relative surface coverage Θ has been calculated from the HS‐AFM images by using a height threshold to distinguish protein‐covered from protein‐free surface areas. As imaging (such as streaks) and image processing (i.e., flattening; see Experimental Section) artifacts may cause variations in the width of the substrate and protein peaks as well as small shifts in the absolute position of the substrate peak (see Figure 2 and Figure S2, S3, Supporting Information), the height distribution function was evaluated for each HS‐AFM image individually. From this evaluation, a height threshold was chosen that lies right between the substrate and the protein peak (see Figure 1d), independent of their absolute positions. The height threshold was then used to separate the protein‐covered (above the threshold) from the protein‐free surface areas (below the threshold). From the thresholded images, the surface coverage Θ was calculated as the ratio of the protein‐covered surface area to the full scan size. As can be seen in the HS‐AFM images shown in Figure 1e, Θ increases with incubation time. Nevertheless, some fluctuations in the calculated surface coverage Θ are observed for consecutive HS‐AFM images, which can be attributed to variations in the applied height thresholds. However, the observed fluctuations are in the range of only ±0.05 and thus should not have a strong impact on the final analysis, in particular in view of the large number of HS‐AFM images recorded over the whole time course (about 100).

**Figure 3:**
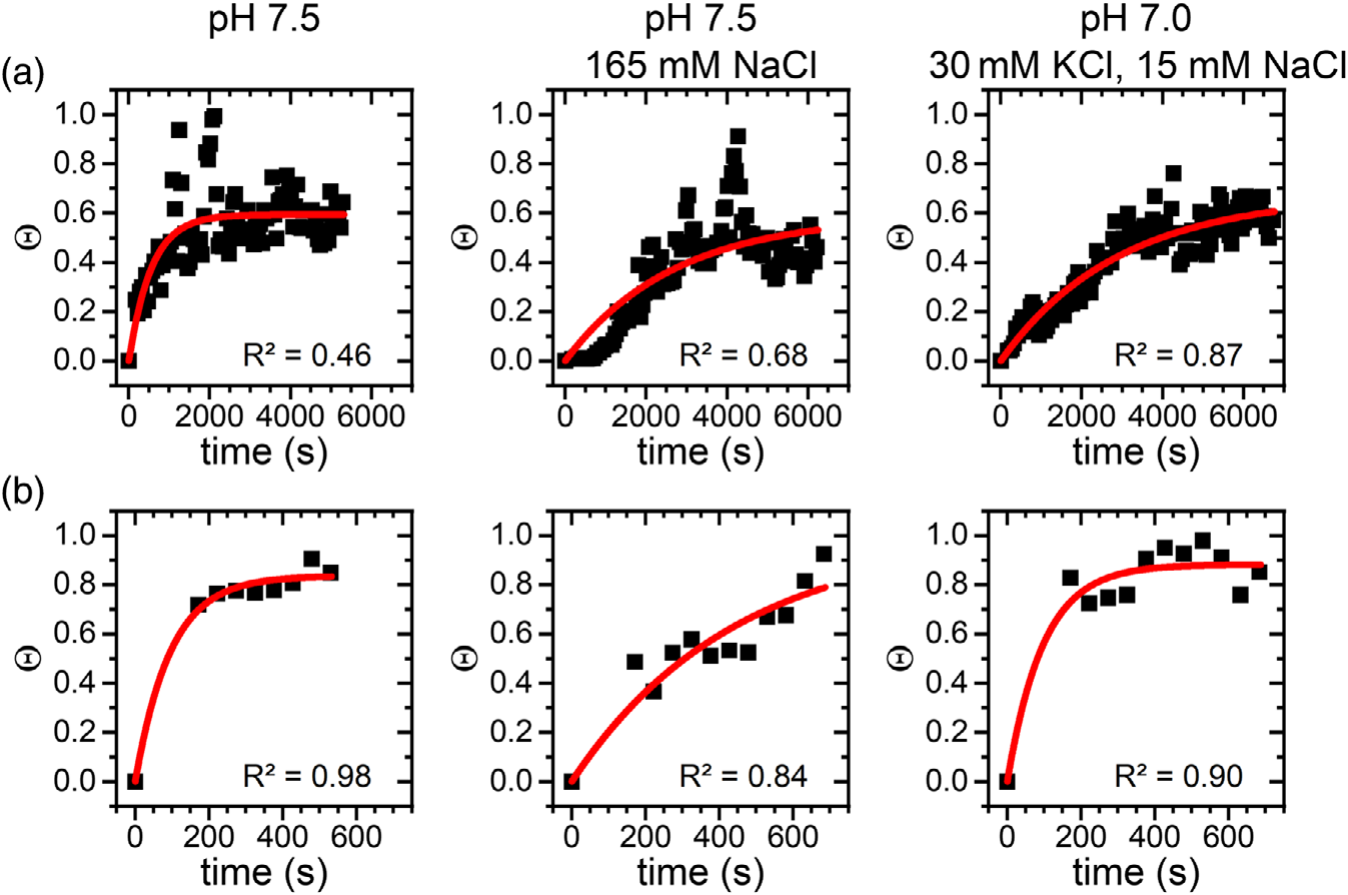
Surface coverage Θ in different electrolytes for the a) Al_2_O_3_(0001) and b) TiO_2_(100) surface as obtained from the HS‐AFM images as a function of time. The solid lines represent exponential fits to the data points according to Equation (1). *R* ^2^ values of the individual fits are given in the panels.

**Figure** **3**a (left panel) shows the time dependence of the so‐obtained protein coverage of the Al_2_O_3_(0001) surface in 10 mM Tris at pH 7.5. Despite rather large fluctuations that can again be attributed to imaging and image processing artifacts as well as sample drift, an overall initial increase in Θ with subsequent saturation is observed. In agreement with the qualitative analysis of the height distribution functions mentioned earlier, the surface coverage saturates after about 2000 s at Θ ≈0.6.

Although a physiological pH of 7.5 was used in this experiment, there were no additional salts. The electrolyte thus differs quite strongly from relevant physiological fluids. Therefore, we have performed additional experiments with electrolyte compositions of 1) 10 mM Tris (pH 7.5) supplemented with 165 mM NaCl and 2) 10 mM Tris (pH 7.0) supplemented with 30 mM KCl and 15 mM NaCl. These electrolyte compositions correspond roughly to that of 1) airway mucosal secretions^[^ ^20^ ^]^ and 2) saliva,^[^ ^21^ ^]^ respectively. As shown in Figure 3a (central and right panel), protein adsorption is visibly slowed down under these conditions and the surface coverage does not reach a stable value within 6000 s of incubation.

The addition of salt results in a shorter Debye length of the electrolyte solution. The observed weaker adsorption thus indicates that adsorption is governed by attractive electrostatic interactions between protein and surface. Indeed, the Al_2_O_3_(0001) surface is negatively charged at pH 7.5,^[^ ^22^ ^]^ whereas the S1 spike protein has a calculated^[^ ^23^ ^]^ isoelectric point (IEP) of 8.3 and should thus carry a small positive net charge at this pH. However, such comparatively large differences in ionic strength may also cause conformational changes in the protein,^[^ ^24^ ^]^ which, in turn, may affect its adsorption behavior.^[^ ^25^ ^]^

To further substantiate that S1 spike protein adsorption at oxide fomites is governed by electrostatic interactions, we have repeated these experiments using a second oxide surface with a different IEP. As shown in Figure 2b, S1 spike protein adsorption at the more negatively charged TiO_2_(100) surface (IEP ≈ 3.5)^[^ ^26^ ^]^ proceeds much faster than for the Al_2_O_3_(0001) surface (IEP ≈ 5.5).^[^ ^22^ ^]^ In fact, in the absence of any salt, the first HS‐AFM image of the series that was recorded after 171.2 s of incubation (see Experimental Section) already shows an almost fully covered TiO_2_(100) surface with the height distribution function being clearly dominated by the protein peak. After 529.6 s of incubation, the low‐z shoulder corresponding to the surface peak has almost completely vanished, indicating the formation of a closed protein film. This strongly increased adsorption at the TiO_2_(100) surface is again in line with electrostatic interactions dominating the adsorption of the SARS‐CoV‐2 S1 spike protein. However, for this surface, ionic strength seems to play a smaller role than for the Al_2_O_3_(0001) surface (see Figure 3b). Although the presence of 165 mM NaCl notably slows down protein adsorption, this is not the case for 30 mM KCl, 15 mM NaCl, and pH 7.0. This may be due to the lower pH, which leads to a higher positive net charge of the protein and may thereby overcompensate the effect of the rather moderate ionic strength. However, TiO_2_ in general also has a larger Hamaker constant than Al_2_O_3_,^[^ ^27^ ^]^ which implies a stronger propensity to participate in van der Waals interactions. The comparably strong adsorption at increased ionic strengths may thus also result from stronger van der Waals interactions between the TiO_2_(100) surface and the protein. It should also be mentioned that due to the rapid adsorption and the limited time resolution in the beginning of the experiment (see Experimental Section), small alterations in adsorption dynamics may not be accessible.

**Figure 4:**
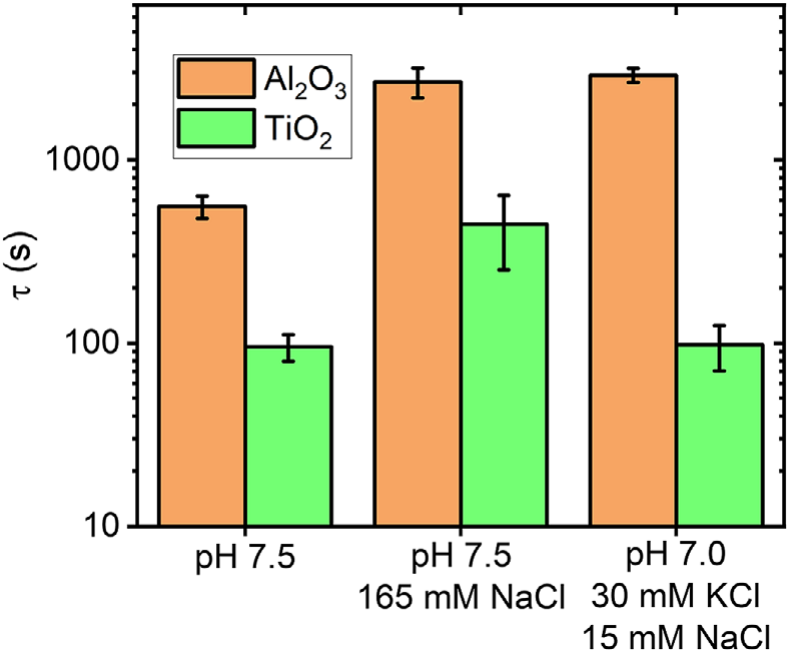
Time constant of adsorption *τ* ± standard error as determined from fitting the data in Figure 3 according to Equation (1). Note the logarithmic *y*‐axis.

To compare protein adsorption kinetics at the different surfaces in a more quantitative way, time constants of adsorption *τ* have been estimated by fitting the time‐dependent surface coverage Θ(*t*) obtained from the HS‐AFM images to the simple exponential function

(1) $$\Theta (t)=\Theta_{s}(1-e^{-\frac{t}{\tau }})$$

with the saturated surface coverage Θ_s_. Even though some of the exponential fits in Figure 3 did not yield very high *R* ^2^ values, we used the so‐obtained time constants as a first‐order approximation to compare adsorption kinetics under the different conditions. Note that logarithmic fits in general resulted in lower *R* ^2^ values than exponential ones. As shown in **Figure** **4**, for all electrolyte conditions investigated in this work, adsorption proceeds about one order of magnitude faster at the more negatively charged TiO_2_(100) surface than at the Al_2_O_3_(0001) surface. Note, however, that adsorption at the TiO_2_(100) surface occurs so fast that we cannot capture the full dynamics (see Figure 3b). The corresponding values in Figure 4 thus represent upper limits, while the actual‐time constants may be even smaller. This in particular concerns conditions 1) pH 7.5 with no salt and 2) pH 7.0 with 30 mM KCl and 15 mM NaCl.

## 3 Conclusion

In summary, we have investigated the adsorption of recombinant SARS‐CoV‐2 spike protein S1 at two different oxide surfaces. This study was enabled by the application of HS‐AFM, which allowed us to use comparatively small protein amounts (5 μg per experiment). Nevertheless, using ultrasmooth, single‐crystalline oxide substrates as model surfaces, we were able to quantitatively evaluate adsorption kinetics by calculating the time‐dependent surface coverage from the HS‐AFM images. To mimic relevant real‐world conditions, we used Al_2_O_3_(0001) and TiO_2_(100) surfaces to model Al‐ and Ti‐based fomite surfaces with native surface oxides. Furthermore, S1 spike protein adsorption was investigated in electrolyte solutions that reflect the pH and major ionic components of airway mucosal secretions and saliva, respectively.

Under all electrolyte conditions, we found much stronger S1 spike protein adsorption at TiO_2_(100) than at the Al_2_O_3_(0001) surface. The stronger adsorption at TiO_2_(100) may be related to its lower IEP and larger Hamaker constant, resulting in stronger electrostatic and van der Waals interactions with the oppositely charged protein. For the Al_2_O_3_(0001) surface, increasing the ionic strength of the electrolyte solution resulted in delayed adsorption. A similar effect was also observed for TiO_2_(100), albeit only at the highest ionic strength. These observations suggest that under these conditions, adsorption of the slightly positively charged S1 spike protein at oxide surfaces is mostly governed by electrostatic interactions.

The S1 spike protein represents the outermost point of the virus envelope, where it is arranged in a complex trimeric structure with the receptor‐binding domains (RBD) facing outward (see Figure 1a,b). The RBD (amino acids 319–541) is also positively charged under the conditions applied in the present experiments and has a similar calculated IEP (≈8.9)^[^ ^23^ ^]^ as the complete S1 protein. We thus expect that electrostatic interactions between the surface and the S1 spike protein and particularly the RBD will also facilitate the initial attachment of complete SARS‐CoV‐2 virions to oxide fomites. After this initial contact, however, other interactions mediated, for instance, by the M protein (IEP ≈ 9.5)^[^ ^28^ ^]^ may become more relevant. Further molecular‐level investigations utilizing different isolated envelope components as well as complete SARS‐CoV‐2 virions are necessary to elucidate the hierarchy of the involved interactions.

In these experiments, we have attempted to mimic relevant biological fluids, i.e., mucus and saliva, with regard to their pH and major ionic components. However, biological fluids are much more complex and contain a large number of different components that have been neglected in the present study, most importantly other proteins. It was recently demonstrated that the presence of proteins increases the stability and viability of SARS‐CoV‐2 at fomite surfaces.^[^ ^8^ ^]^ In such a setting, the additional protein components may compete with the spike proteins and complete SARS‐CoV‐2 virions for free adsorption sites at the fomite surfaces and thereby modulate the relative contributions of the involved interactions. Even though competitive protein adsorption is rather well studied for plasma proteins, the effects on the overall adsorption behavior are typically highly complex and difficult to predict.^[^ ^29^ ^]^ Again, further studies are needed to shed light on the molecular mechanisms governing SARS‐CoV‐2 adsorption at fomite surfaces in complex physiological media.

## 4 Experimental Section

4.1

4.1.1

##### Substrate Surface Preparation

Epi‐ready Al_2_O_3_(0001) and TiO_2_(100) substrates (1 × 1 × 0.5 cm^3^) were purchased from Crystal GmbH. The substrates were cleaned with ethanol and HPLC grade water (VWR) and subsequently dried under a stream of ultrapure air. To remove organic contaminations, the substrates were then treated with an O_2_ plasma (diener Zepto, diener electronic) for 30 s. Afterward, the substrates were immediately immersed in 1 mL of the corresponding buffer solution. The substrate surfaces were then evaluated in this solution by HS‐AFM (see later). If contaminants were still observed in the HS‐AFM images, the cleaning process would be repeated until obtaining a clean surface (see Figure S1, Supporting Information).

##### Protein Sample Preparation

Lyophilized recombinant SARS‐CoV‐2 S1 spike protein (0.1 mg) (amino acids 16–685 with C‐terminal His‐Tag) was purchased from Acro Biosystems, reconstituted in 167 μL HPLC grade water (VWR) to yield a 600 μg mL^−1^ stock solution, and stored at −70 °C until further use. HS‐AFM measurements were performed with 5 μg mL^−1^ SARS‐CoV‐2 S1 spike protein in three different electrolyte solutions: 1) 10 mM Tris (Sigma‐Aldrich) at pH 7.5; 2) 10 mM Tris at pH 7.5 with 165 mM NaCl (VWR Chemicals); and 3) 10 mM Tris at pH 7.0 with 30 mM KCl (Merck) and 15 mM NaCl.

##### HS‐AFM Imaging

HS‐AFM imaging was performed using a JPK NanoWizard ULTRA Speed and USC‐F0.3‐k0.3 cantilevers (NanoWorld). Directly following the measurement of the cleaned substrate surface (corresponding to incubation for 0 s), 0.5 mL of the working buffer was removed from the liquid cell and replaced by 0.5 mL protein‐containing solution. This whole procedure took close to 2 min, so that the first HS‐AFM scan in the presence of the S1 spike protein was initiated 120 s after the injection of SARS‐CoV‐2 S1 protein. HS‐AFM images were recorded with 1 × 1 μm^2^ scan size, 10 Hz line rate, and 512 × 512 px^2^ resolution, corresponding to 51.2 s per frame.

##### Statistical Analysis

The HS‐AFM images were batch‐processed in the JPKSPM Data Processing software by 1) subtracting a third degree polynomial fit from each scan line independently, 2) subtracting a first degree polynomial fit from each scan using a limited data range between 0% and 70%, 3) replacing outliers with the median of neighboring pixels (mask shape: constricted square), 4) subtracting a first degree polynomial surface from the image, and 5) replacing lines in the image by interpolating. Each preprocessed image was then analyzed individually in Gwyddion version 2.52,^[^ ^30^ ^]^ after setting the pixel with the lowest height value to zero. The height distribution function of each recorded HS‐AFM image was calculated using the Statistical Functions tool and evaluated for each image individually to choose a height threshold located between the substrate and the protein peaks independent of their absolute center positions. Height thresholds were applied to the HS‐AFM images using the Mark by Threshold tool, and the surface coverage Θ was calculated using the Grain Summary tool. All further analyses including the exponential fits of the time‐dependent Θ values according to Equation (1) (see Figure 3) were done in OriginPro 2020. The time constants of adsorption in Figure 4 are given as the fit‐derived value ± standard error.

## Conflict of Interest

The authors declare no conflict of interest.

## Supporting information

Supplementary Material

Supplementary Material

Supplementary Material

Supplementary Material

Supplementary Material

Supplementary Material

Supplementary Material
