# Supplementary material for: Adsorption of SARS‐CoV‐2 Spike Protein S1 at Oxide Surfaces Studied by High‐Speed Atomic Force Microscopy
Source: Adv Nanobiomed Res. 2020 Dec 18;1(2):2000024. doi: 10.1002/anbr.202000024 (PMC7883093; doi:10.1002/anbr.202000024)
Supplement: Supplementary file 1 — Supplementary Material [file ANBR-1-2000024-s001.pdf]

Copyright WILEY-VCH Verlag GmbH & Co. KGaA, 69469 Weinheim, Germany, 2018.

Supporting Information

Adsorption of SARS-CoV-2 Spike Protein S1 at Oxide Surfaces Studied by High-Speed Atomic Force Microscopy

*Yang Xin, Guido Grundmeier, and Adrian Keller**

HS-AFM images of the pristine Al_2O_3(0001) and TiO_2(100) surfaces

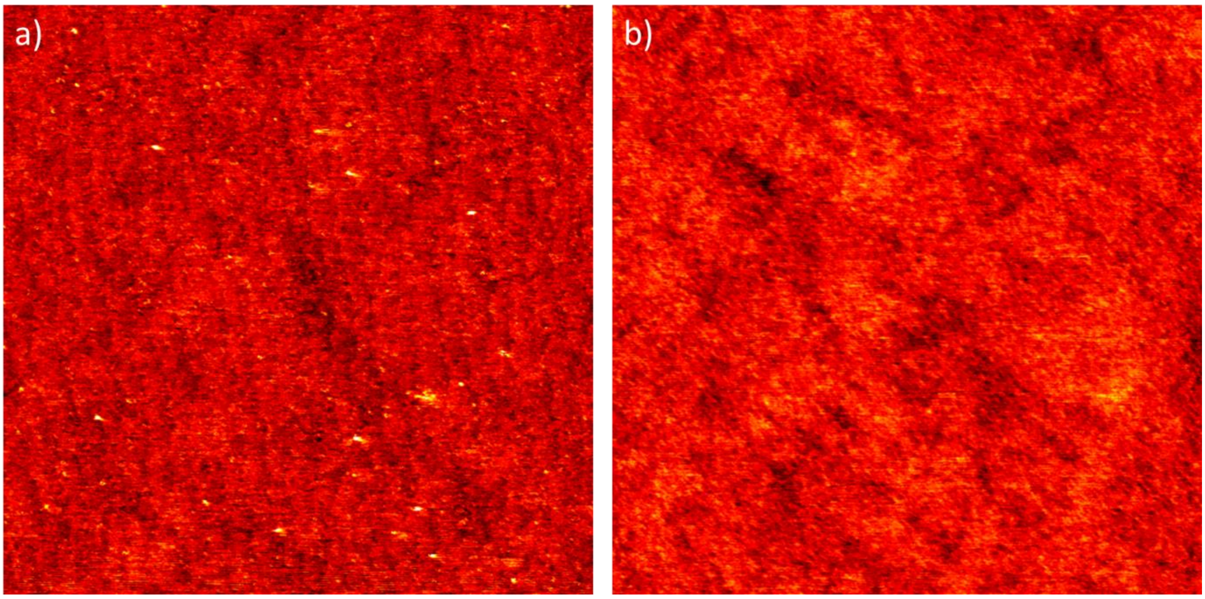

Figure S1. HS-AFM images of an Al_2O_3(0001) (a) and a TiO_2(100) substrate (b) in 10 mM Tris (pH 7.5) without proteins. The images have a size of 1 x 1 µm² and a height scale of 2 nm.

Additional AFM images and height distribution functions

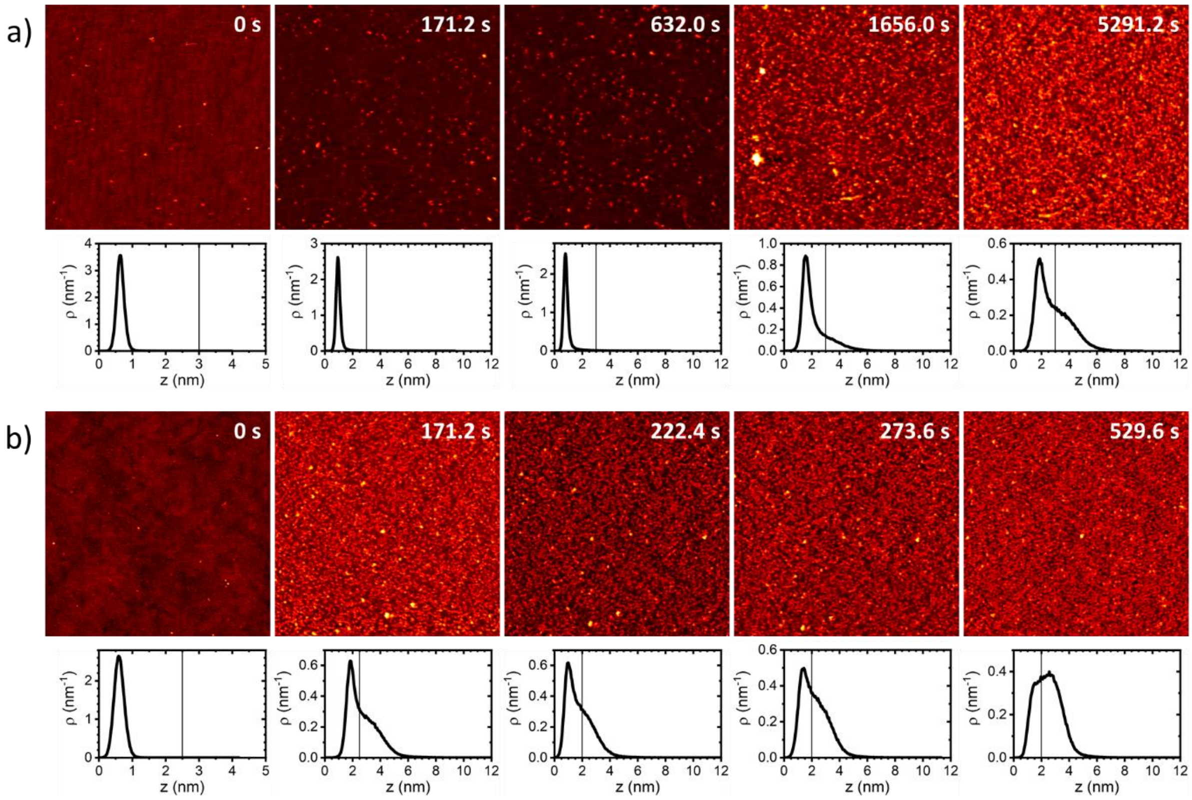

Figure S2. HS-AFM images (1 x 1 µm²) of SARS-CoV-2 spike protein S1 in 10 mM Tris (pH 7.5) with 165 mM NaCl adsorbed to (a) an Al_2O_3(0001) and (b) a TiO_2(100) surface recorded at different time points as indicated. Height scales are 5 nm for the clean substrate surfaces at 0 s and 12 nm for the protein covered surfaces at later time points. Below the HS-AFM images, the corresponding height distribution functions are depicted. The vertical lines in the plots represents the height thresholds applied in the statistical analyses.

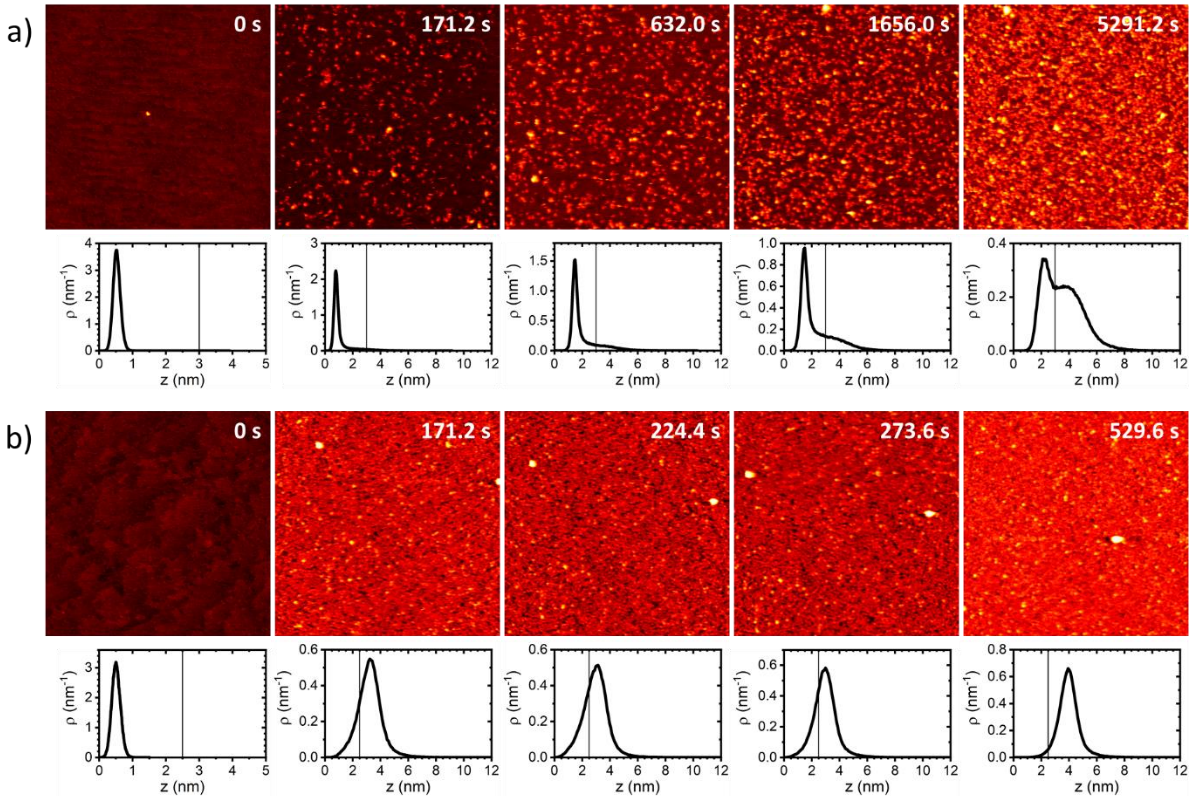

Figure S3. HS-AFM images (1 x 1 µm²) of SARS-CoV-2 spike protein S1 in 10 mM Tris (pH 7.0) with 30 mM KCl and 15 mM NaCl adsorbed to (a) an Al_2O_3(0001) and (b) a TiO_2(100) surface recorded at different time points as indicated. Height scales are 5 nm for the clean substrate surfaces at 0 s and 12 nm for the protein covered surfaces at later time points. Below the HS-AFM images, the corresponding height distribution functions are depicted. The vertical lines in the plots represents the height thresholds applied in the statistical analyses.

Evaluation of tip effects

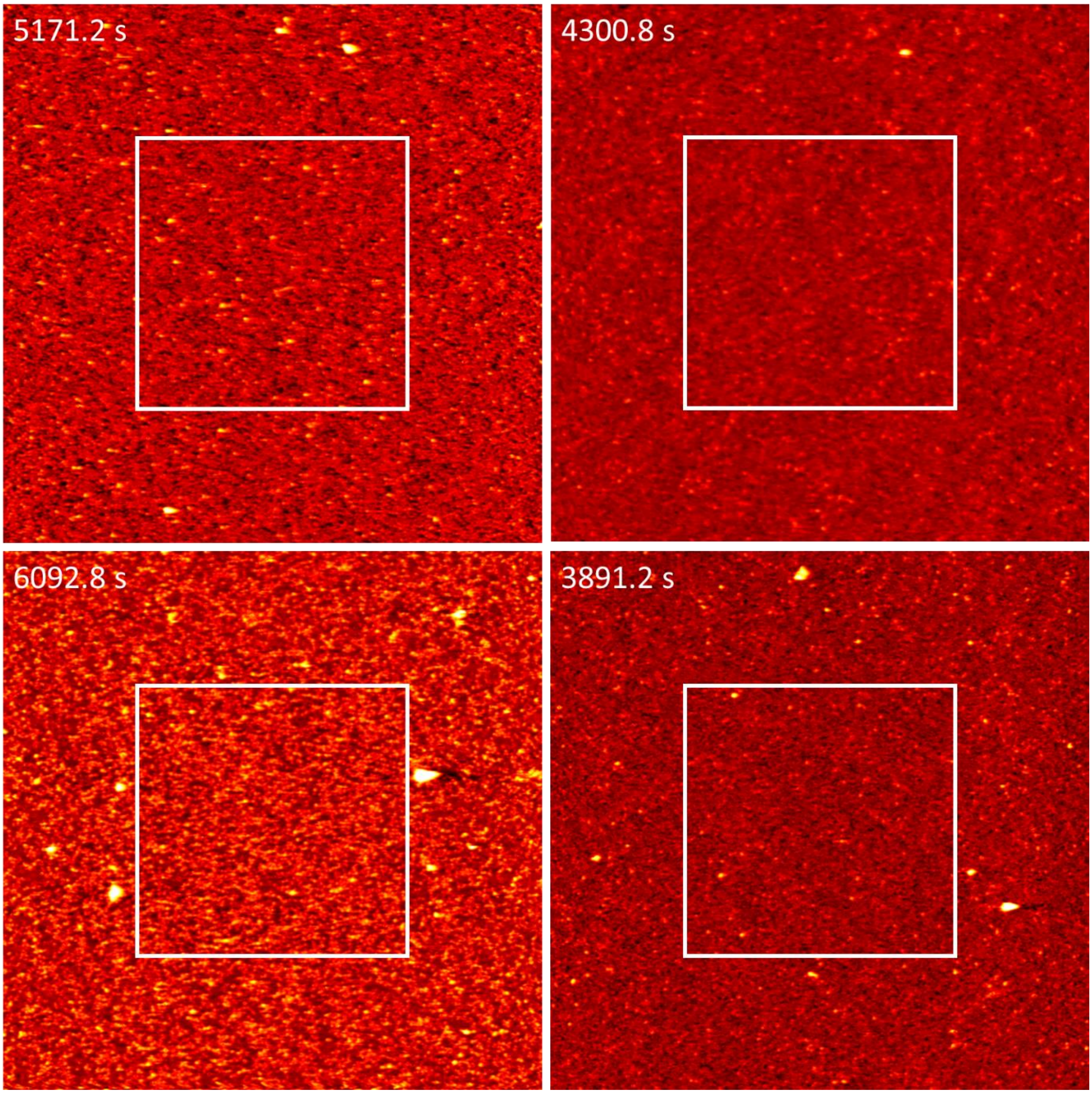

Figure S4. Zoomed-out HS-AFM images recorded after protein adsorption at Al_2O_3(0001) (left) and TiO_2(100) surfaces (right) in 10 mM Tris (pH 7.5) with (lower row) and without 165 mM NaCl (upper row). The images have a size of 2 x 2 µm² and a height scale of 12 nm. The white squares indicate the 1 x 1 µm² areas that have been continuously scanned during the experiments for the times indicated in the images. In neither image, an influence of the scanned tip can be noticed.
